# Biomechanical Characterisation of Gait in Older Adults: A Cross-Sectional Study Using Inertial Sensor-Based Motion Capture

**DOI:** 10.3390/bioengineering12080889

**Published:** 2025-08-20

**Authors:** Anna Letournel, Madalena Marques, Ricardo Vigário, Carla Quintão, Cláudia Quaresma

**Affiliations:** 1 Departamento de Ciências Biomédicas, Instituto Politécnico de Setúbal, Escola Superior de Saúde, Campus do Instituto Politécnico de Setúbal, Estefanilha, Edifício ESCE/ESS, 2914-504 Setúbal, Portugal; 2 Instituto de Biofísica e Engenharia Biomédica, Faculdade de Ciências da Universidade de Lisboa, 1749-016 Lisboa, Portugal; 3 Physics Department, NOVA School of Science and Technology, NOVA University of Lisbon, 2829-516 Caparica, Portugalcmquintao@fct.unl.pt (C.Q.); q.claudia@fct.unl.pt (C.Q.); 4 Laboratory for Instrumentation Biomedical Engineering and Radiation Physics, NOVA School of Science and Technology, NOVA University of Lisbon, 2829-516 Caparica, Portugal; 5 Associated Laboratory in Translation and Innovation Towards Global Health, NOVA School of Science and Technology, NOVA University of Lisbon, 2829-516 Caparica, Portugal

**Keywords:** older adults, gait analysis, joint kinematics, ageing and mobility, wearables

## Abstract

The ageing of the global population, especially in developed countries, is driving significant societal changes. In Portugal, demographic data reflect a marked increase in the ageing index. Understanding gait alterations associated with ageing is essential for the early detection of mobility decline and fall risk. This study aimed to analyse gait patterns in older adults to contribute to a biomechanical ageing profile. Thirty-six community-dwelling older adults (29 female, 7 male; mean age: 74 years) participated. Gait data were collected using the Xsens full-body motion capture system, which combines inertial sensors with biomechanical modelling and sensor fusion. Spatiotemporal and kinematic parameters were analysed using descriptive statistics. Compared to younger adult norms, participants showed increased stance and double support phases, reduced swing phase, and lower gait speed, stride length, and cadence, with greater step width. Kinematic data showed reduced peak plantar flexion, knee flexion, and hip extension, but increased dorsiflexion peaks—adaptations aimed at stability. Despite a limited sample size and lack of clinical subgroups, results align with age-related gait literature. Findings support the utility of wearable systems like Xsens in capturing clinically relevant gait changes, contributing to normative biomechanical profiling and future mobility interventions.

## 1. Introduction

Older adults face a unique set of challenges related to both physical and mental health, which must be addressed to ensure a higher quality of life as they age. As the global population continues to age, these challenges are expected to increase the demand for primary and long-term healthcare services. Furthermore, there will be a need for a larger, better-trained healthcare workforce and the adaptation of living spaces to accommodate older adults’ needs [1].

Given the increasing life expectancy, it is essential for society to adapt to the rapid ageing process. One of the first steps in this adaptation is to develop a comprehensive understanding of the ageing profile of the population. A clear understanding allows for the development of policies, procedures, and infrastructure that can effectively meet the demands of an ageing society.

Research into the ageing process must address a variety of factors that affect the everyday lives of older individuals. These include the perceived quality of life, cognitive function, subjective happiness, speech patterns, and eating habits. However, one critical aspect that has often been overlooked in previous studies is the biomechanical changes associated with ageing, specifically in relation to gait patterns. Gait analysis is a key area for understanding how age-related changes affect mobility and independence. In particular, spatiotemporal parameters and the kinematics of key joints such as the hip, knee, and ankle can provide valuable insight into an individual’s overall physical condition [2,3].

Several studies have already examined gait changes in older adults using laboratory-based tools such as optical motion capture systems, pressure-sensitive walkways, or force platforms [4,5,6,7]. These studies have provided key insights into spatiotemporal variability, stability, and fall risk. More recently, wearable inertial measurement units (IMUs), such as the Xsens system, have become increasingly adopted for gait analysis due to their portability, low cost, and ability to capture motion data in naturalistic environments [8,9]. These technologies provide a practical solution for studying gait outside the laboratory, particularly in ageing populations.

In addition to IMUs, various advanced technologies have been developed for gait monitoring. These include AI-assisted insole sensing systems that enable multifunctional plantar-healthcare applications, pressure-sensitive mats, and wearable smart textiles embedded with sensors [10,11,12]. Such systems provide complementary insights by capturing detailed plantar pressure distribution and subtle gait pattern changes, allowing continuous real-world monitoring [13,14]. Despite these advances, balancing accuracy, portability, and user comfort remains challenging, underscoring the relevance of IMU-based systems like Xsens for comprehensive gait analysis in ageing populations.

Although previous studies have explored various aspects of ageing—such as the Study of the Ageing Profile of the Portuguese Population, which assessed health perceptions and cognitive decline among older adults—they have not focused on biomechanical aspects like gait analysis [15]. This represents a significant gap, especially considering that the ability to walk, and the quality of one’s gait, are essential for maintaining independence and preventing falls, which are major concerns among older adults [16,17].

The primary aim of this study is to analyse the biomechanical parameters of gait in older individuals, with a focus on spatiotemporal factors and joint kinematics. The study specifically examines the movement patterns of the hip, knee, and ankle joints during walking using a portable IMU-based system. To the best of our knowledge, this is the first study to characterise gait biomechanics in the Portuguese ageing population using the Xsens system. Understanding these parameters in detail will contribute to a broader knowledge of the biomechanical changes that occur with ageing and may offer insights into interventions that could improve mobility and reduce the risk of falls [18].

While this research focuses on a specific group of older adults, the methodologies used and the findings obtained are expected to be applicable to other populations with similar characteristics. This study aims to contribute to the development of tailored responses to the needs of older adults, enhancing their quality of life through better-informed healthcare and support services.

By providing a deeper understanding of the biomechanics of gait in older adults, this research can help guide future interventions designed to improve mobility, enhance independence, and reduce the risk of injury, ultimately improving the overall well-being of this population.

## 2. Materials and Methods

This study investigates the biomechanical parameters of gait in older adults using a full-body motion capture system equipped with inertial measurement unit (IMU) sensors to assess motor function and mobility alterations associated with ageing. The system provides detailed data on spatiotemporal and kinematic aspects of the gait cycle, enabling precise analysis of joint movement and stability patterns.

### 2.1. Participants

Participants were recruited from daycare centres located in both urban and rural settings to obtain a representative and heterogeneous sample reflecting distinct lifestyle factors. The urban cohort originated from a densely populated metropolitan area, while the rural cohort was drawn from a sparsely populated region. It was hypothesized that rural participants engage in higher levels of physical activity due to environmental and occupational demands, whereas urban participants may have increased cultural and social activity but lower physical activity levels. This stratified recruitment approach aimed to capture potential variations in gait biomechanics attributable to differing environmental and lifestyle influences.

The study sample consisted of 36 older adults (29 females, 7 males), aged 62 to 91 years (mean ± SD: 74 ± 7 years). Exclusion criteria comprised cognitive impairment limiting the ability to provide informed consent, diagnosed dementia, presence of lower limb prostheses, history of lower limb surgery, anatomical malformations or functional impairments affecting gait, and reliance on walking aids.

### 2.2. Ethics

The study protocol received approval from the Comissão Especializada de Ética em Investigação (Approval Number: 67), and all participants provided written informed consent prior to participation in accordance with the Declaration of Helsinki.

### 2.3. Experimental Procedure

Biomechanical data were collected using Xsens equipment and dedicated software as well as a six-minute walk test. The protocol involved the following steps:

- Anthropometric measurements
- Non-dominant hand grip strength measurement
- Six-minute walk test (6 MWT)
- Placement of wearable sensors
- Calibration
- Data recording

*Anthropometric and Hand Grip Strength Measurements* Anthropometric measurements were taken for each participant. Height was measured using a stadiometer (cm), and weight was recorded with an analogue scale (kg). Foot size, arm span, ankle height, hip height, knee height, and shoulder width were measured using a tape measure. Hand grip strength was assessed with a hydraulic hand dynamometer, recording three trials to calculate an average.

*Six-Minute Walk Test* Participants completed the 6 MWT by walking at a self-selected pace along a 25 m unobstructed walkway. The total distance covered during six minutes was recorded, and walking speed was calculated based on the number of laps completed. Participants were encouraged to walk as far as possible, with periodic time updates provided by the researcher. Rest breaks were allowed as needed.

*Measurement Protocol and Wearable Sensors Placement* All gait assessments were conducted using the Xsens MVN system, which consists of 17 wireless inertial measurement units (IMUs) placed on anatomical landmarks according to the manufacturer’s recommended full-body configuration.

**Figure 1:**
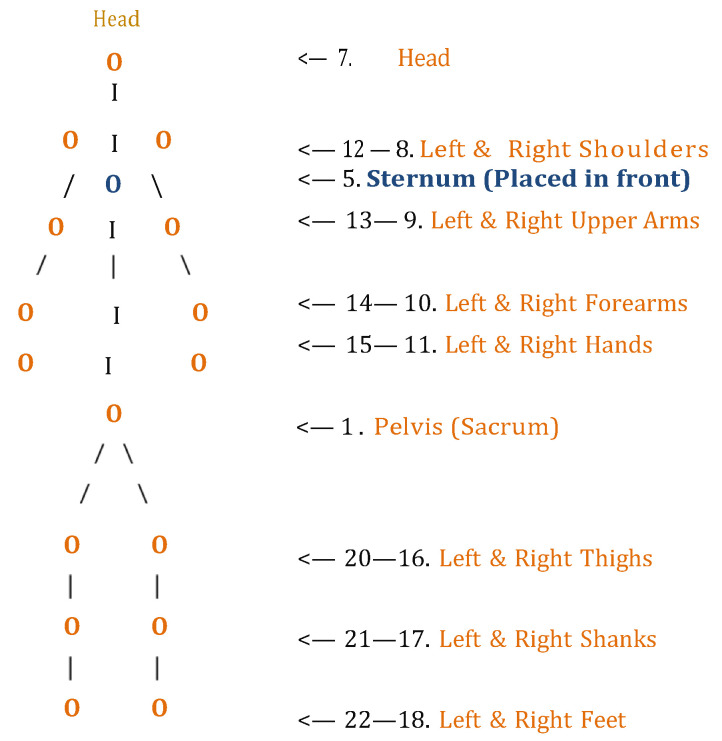
Line-based schematic of the human body viewed from the back, illustrating the placement of 17 Xsens MVN sensors and its attributed numbering. All sensors are shown on the posterior side except for a single sensor located on the sternum (anterior view).

The 17 IMUs were positioned at the following anatomical locations: head, upper back (T3), lower back (L5), pelvis (sacrum), left and right upper arms, left and right forearms, left and right hands, left and right thighs (mid-femur), left and right shanks (mid-tibia), and left and right feet (dorsal surface). Sensors were placed on prominent bony landmarks such as the hip (greater trochanter), knee (medial epicondyle), and ankle (lateral malleolus). Sensor locations were fine-tuned in the software prior to calibration by measuring the distances between sensors and the corresponding anatomical landmarks to enhance accuracy as shown in Figure 1.

Sensors were secured using elastic straps with hook-and-loop closures to ensure stability during walking trials, positioned close to the joints without restricting movement. The sensors were positioned near the joints without restricting movement. Sensor locations were corrected in the software by measuring the distance between the sensors and the anatomical landmarks prior to calibration.

Participants walked barefoot at a self-selected pace along a straight, unobstructed 7 m path while the Xsens system recorded kinematic data.

*Xsens Walking Trials* Walking speed during these trials was derived from the spatiotemporal data captured by the system. Due to differences in protocol length and purpose, walking speeds obtained from the 6 MWT and Xsens trials may differ. Specifically, the 6 MWT measures endurance walking speed over a prolonged duration, encouraging maximal effort, whereas the Xsens trials assess natural, short-distance gait patterns optimised for detailed biomechanical. Additionally, the controlled laboratory environment during Xsens assessments may promote more cautious and steady walking compared to the sustained, maximal effort encouraged in the 6 MWT.

Participants walked along a 7 m straight walkway during Xsens trials. Although short walkways may include initial acceleration and deceleration phases that could influence spatiotemporal metrics, the central portion of each trial was prioritized for analysis. Participants were instructed to start walking before entering the measurement zone and to continue slightly beyond it, ensuring that the data used reflected near steady-state gait. This approach follows recommendations from previous studies demonstrating that short walkways can still yield reliable gait parameter estimates when careful data processing is applied [8,9].

*Calibration and Data Recording* Calibration was performed with participants standing in an anatomical (N-pose) position for 30 s. Calibration quality was assessed by the software on a scale from “good” to “fail,” and only trials rated as “good” or “acceptable” were included for analysis. Data were recorded at an update rate of 120 Hz, beginning with one minute of static data followed by five one-minute motion trials. During motion trials, participants walked back and forth along a seven-meter hallway at a regular pace. To ensure optimal battery performance and data acquisition quality, recordings were conducted in two-hour sessions involving multiple participants.

### 2.4. Data Processing and Analysis

Data processing was conducted using Xsens MVN Studio and Visual3D software. Following initial system calibration, static and dynamic motion trials were applied to the Xsens musculoskeletal model, which provides direct outputs of joint kinematics and linear velocity data [8,9]. Prior to data collection, participants performed a short familiarisation trial to adapt to the Xsens system and walking in the laboratory environment, reducing the potential for altered gait patterns due to novelty.

Raw linear velocity time series along the mediolateral (x), anteroposterior (y), and vertical (z) axes were extracted for further analysis. To attenuate high-frequency noise while preserving critical gait characteristics, signals were filtered using a bidirectional, fourth-order low-pass Butterworth filter with a cut-off frequency of 6 Hz, in line with established biomechanical filtering protocols [19,20]. This cut-off frequency was selected based on literature standards and visual inspection of pilot signals to ensure relevant gait dynamics were preserved.

The vertical component (z-axis) of the velocity signal was utilised to manually identify key gait events, specifically right toe-off, right heel strike and left toe-off, left heel strike. Manual identification was performed by a trained operator; however, no formal intra- or inter-rater reliability tests were conducted. These manually annotated events were subsequently used to develop and validate pattern recognition algorithms that automated gait event detection across all motion trials [21,22]. No gold-standard validation (e.g., force plates) was performed; thus, algorithm accuracy relies on the manually annotated dataset.

Step width was calculated based on these manually annotated foot strikes, including both heel and toe contact, as determined from the Xsens foot sensors.

All sensors were placed by a single trained operator according to the standard Xsens protocol, with careful alignment over anatomical landmarks to minimise soft tissue artefact and inter-participant variability. From the filtered and event-annotated data, joint kinematic variables—including angular amplitudes and angular velocities of the hip, knee, and ankle—were extracted. Key parameters included maximum flexion/extension angles, peak angular velocities, and the temporal occurrence of these peaks within the gait cycle. Complementary spatiotemporal gait metrics, such as gait speed, step length, step time, stance time, swing time, and double limb support time, were also computed [23].

Data underwent manual quality control to identify and correct artefacts and errors. Notably, ankle joint data required angular correction due to sensor placement over the tarsus at an approximate 15º angle to maintain biomechanical accuracy. Following correction, residual noise was further reduced using a moving average filter with a window size of five samples [24].

Statistical analyses were performed using SPSS Statistics (version 27), incorporating descriptive statistics (mean, standard deviation, variance, minimum, and maximum) and peak-to-peak amplitude computations to characterise biomechanical parameters.

**Figure 2:**
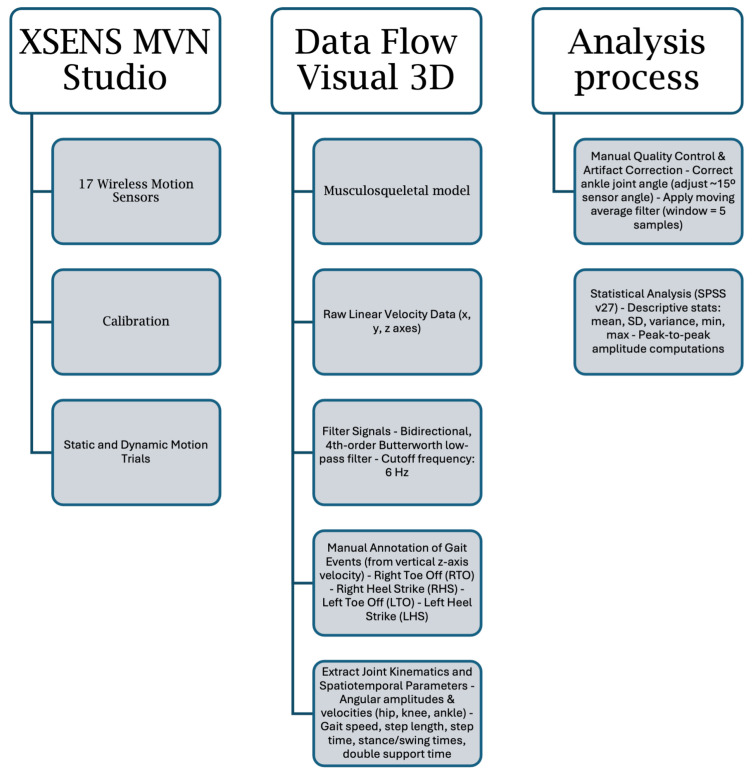
Block diagram of the procedure including calibration, signal filtering, manual gait event detection, kinematic and spatiotemporal parameter extraction and statistical analysis.

The block diagram illustrating the data processing pipeline for gait analysis using Xsens MVN Studio and Visual3D is presented in Figure 2.

## 3. Results

**Figure 3:**
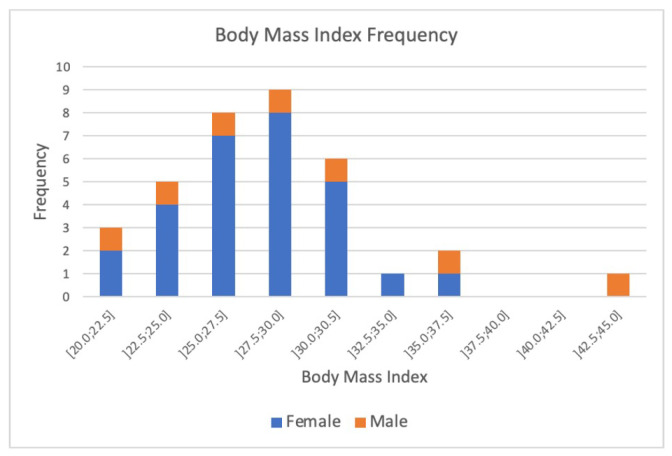
Distribution of body mass index (BMI) values among the study population. The histogram illustrates the frequency of participants across BMI categories, highlighting the prevalence of overweight and obesity within the cohort.

The sample under study has an average body mass index (BMI) of 28 kg/m^2^, with 8 individuals classified as normal weight, 18 as overweight, and 10 as obese. As seen in Figure 3, the sample is not entirely homogeneous in terms of BMI.

**Figure 4:**
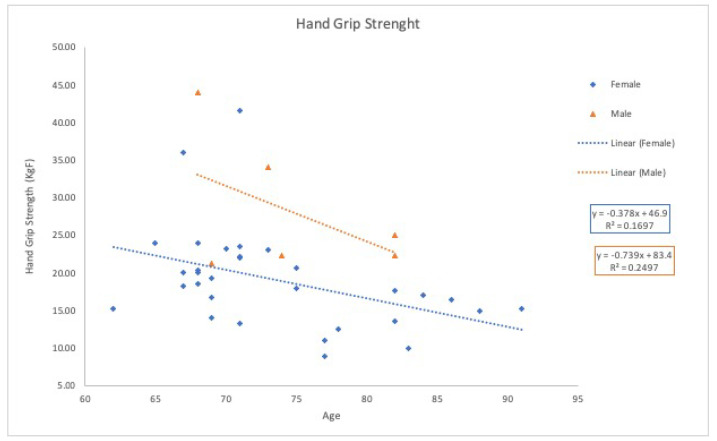
Hand Grip Strength variation, as a function of age for both female and male participants.

The population’s mean grip strength is (21 ± 8) kgf. Males exhibit an average grip force of (28 ± 8) kgf, while females show (19 ± 7) kgf. Figure 4 illustrates the expected age-related decline in hand grip strength.

**Figure 5:**
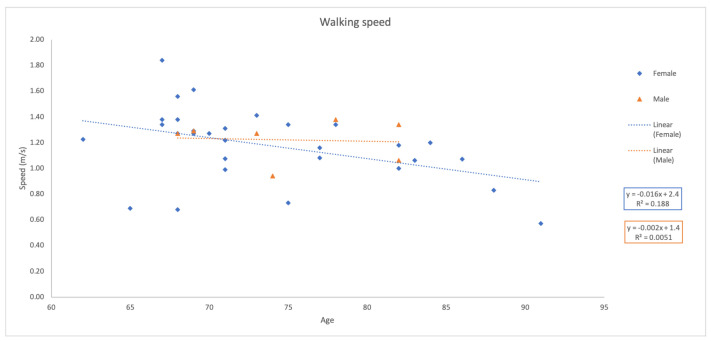
Representation of the variation of the walking speed with age, for both female and male volunteers, using data from the six minute walk test.

Walking speed was assessed using the 6-Minute Walk Test, with results shown in Figure 5. As expected, walking speed decreases with age [25] and aligns with values reported by Imura et al. (2007) [26]. Figure 5 further suggests a greater decline in walking speed for females, though conclusions are limited due to the small male representation.

However, the resulting coefficients of determination (R^2^) were low or near zero, indicating that the relationship between age and these functional measures is poorly described by a linear model. This suggests that age-related changes in hand grip strength and walking speed follow more complex, potentially nonlinear trends.

Additionally, walking speed was calculated using Xsens data, yielding an average of 1.2 ± 0.2 m/s for the six-minute walk test and 0.5 ± 0.2 m/s for Xsens. The disparity indicates that sensor use affects natural gait, at least in terms of speed, preventing direct comparison of absolute values.

Table 1 presents mean results for stance phase, swing phase, and gait cycle (GC) across the population. Inter-subject variability for gait cycle phase percentages was not explicitly calculated; however, visual inspection suggested that variability across participants was low. The stance phase comprises 82% of the GC, while the swing phase accounts for 18%. Compared to adults, where stance and swing phases represent 60% and 40% of the GC, respectively [27], older adults exhibit an increased stance phase and a reduced swing phase.

The mean stride length is (1.1 ± 0.2) m, with a duration of (2.2 ± 0.5) s. For adults without gait pathology, stride length is approximately 1.41 m [28]. The mean step width is (0.2 ± 0.1) m. Step width values were calculated based on manually annotated foot strikes, including both heel and toe contact, which explains the slightly higher values compared to literature norms (0.08–0.10) m [28]. Left cadence averages (88 ± 20) steps per minute, and right cadence (97 ± 16) steps per minute, compared to the adult mean of 113 steps per minute [28].

To characterise joint angular motion, peak flexion and extension angles of the hip and knee, as well as peak plantar flexion and dorsiflexion angles of the ankle, were analysed as shown in Table 2. Compared to reference adult values, no differences were observed in maximum hip flexion. However, hip extension amplitude decreased by approximately 30% [28].

Regarding the knee joint, peak knee flexion was lower than the adult reference value of 60º, while maximum knee extension remained consistent with adult values of 5% (Perry, 2010). For the ankle, a reduction in mean maximum plantar flexion (average 20º) and an increase in peak dorsiflexion (average 5º) were observed, suggesting reduced plantar flexion and increased dorsiflexion compared to adults [28].

Overall, these findings highlight significant age-related changes in gait parameters, including reduced walking speed, altered stance-to-swing phase ratios, and modifications in joint movement patterns. These changes contribute to decreased mobility and stability in older adults, reinforcing the importance of gait analysis in ageing research.

The findings indicate that the studied population is, on average, overweight, consistent with the BMI distribution where most participants were classified as overweight or obese. This aligns with global ageing trends that associate higher BMI with older age groups [29].

Moreover, grip strength analysis revealed an age-related decline, with men generally showing higher values than women. However, the grip strength values for men in this study were slightly lower than those reported in previous literature [30], possibly reflecting broader population ageing trends.

Gait symmetry was preserved between limbs in the study population, consistent with earlier findings by Oberg et al. (1994) [31]. Nevertheless, results showed an increased stance phase duration and a concomitant reduction in swing phase, a characteristic gait adaptation in ageing [5]. The swing phase plays a critical role in limb propulsion, and its shortening may reflect diminished muscle strength and reduced limb advancement [32]. In contrast, prolongation of the stance phase is likely a compensatory adaptation to enhance stability, as reduced postural control is common among older adults [33,34].

Typically, the double support phase occupies approximately 20% of the gait cycle in healthy adults [35]. In this study, it was prolonged to 53%, highlighting an increased need for stability. Prolonged double support is frequently observed in older adults with compromised balance and is considered an adaptive mechanism to reduce fall risk [16,26].

Walking speed decreased when using the Xsens sensors, although the equipment does not directly restrict motion. This discrepancy may stem from the novelty of the equipment or heightened self-awareness during data collection [9]. The six-minute walk test (6 MWT), which allows subjects to achieve a more natural walking rhythm over a longer period, likely better represents habitual gait. Thus, factors such as sensor unfamiliarity, trial design, and participant awareness may all have contributed to the observed lower speeds during Xsens trials.

The observed decrease in walking speed aligns with prior findings that attribute this to reduced stride length with ageing [36]. Our data showed decreased stride length and increased stride duration, reflecting trends associated with age-related sarcopenia and neuromuscular degradation [5,25].

Step width values were slightly higher than those typically reported for older males (0.10 m) and females (0.079 m) [16]. Since increased step width is associated with greater lateral stability, this may reflect a compensatory mechanism to enhance balance [37].

Cadence was lower than the reference adult value of 113 steps per minute [38], consistent with evidence that cadence declines with age due to reductions in neuromuscular efficiency and balance control [5,39].

Hip joint kinematics showed preserved flexion but reduced extension amplitude compared to adult reference values [31], potentially contributing to shorter stride length and reduced walking speed. Alternatively, this reduction may reflect a conscious strategy to maintain balance [40].

For the knee, peak flexion was lower than adult norms, while extension remained unchanged [31]. Reduced flexion may be due to joint stiffness or weakened push-off forces, which could also be influenced by reduced plantar flexion at the ankle.

Ankle kinematics revealed decreased plantar flexion and increased dorsiflexion, diverging from adult reference values [31]. The reduced plantar flexion may reflect lower push-off capability due to muscle loss, while the increased dorsiflexion could indicate a compensatory adaptation for postural stability [34].

No significant gender differences were found in joint angular motion, suggesting that age-related biomechanical changes affect both sexes similarly, as also reported in prior studies [41].

Muscle strength loss, which closely affects joint dynamics, tends to decline with age due to reductions in muscle mass and fibre number. Research indicates a 20–40% strength reduction by age 70 [36], consistent with trends observed in this study. Although muscle mass was not directly assessed, it likely contributed to the observed gait adaptations.

Several limitations must be acknowledged in this study. Joint angles were derived indirectly from inertial sensor data, which introduces potential errors related to sensor placement and soft tissue artefacts. All sensors were placed by a single trained operator according to standard Xsens protocol to minimise inter-participant variability, but residual biases in joint kinematics may remain. Although standardised calibration procedures and specific corrections (e.g., adjustment for ankle sensor angle) were implemented to minimise these effects, residual biases in joint kinematics may remain. The 15° ankle correction was determined empirically, as recommended by prior literature and visual inspection of pilot data. In addition, the 6 Hz cut-off frequency used for low-pass filtering was adopted from prior literature on IMU-based gait analysis in older adults, rather than being optimised for the present dataset, which may limit the potential for dataset-specific signal refinement. Pilot inspections confirmed preservation of gait-relevant features, but dataset-specific optimisation was not performed.

Moreover, gait events such as heel strike and toe-off were identified manually using the vertical velocity signal, without formal intra- or inter-rater reliability testing and without concurrent validation against a gold-standard force plate. Although force plates were available in our laboratory, their installation adjacent to a metal grid introduced electromagnetic interference that affected signal reliability. We therefore opted not to use these data and instead developed a Visual3D processing pipeline for manual event annotation, ensuring consistent detection across participants. While this method is inherently more subjective, previous studies have shown that IMU-based event detection can achieve acceptable accuracy when carefully implemented [8,9]. Nonetheless, the absence of validated force plate measurements limits the precision of gait event detection and may affect the accuracy of spatiotemporal parameters. Additionally, pattern recognition algorithms for automated gait phase detection were not validated against standard datasets.

Despite these limitations, the Xsens system offers portability and cost-effectiveness, enabling gait assessments in ecologically valid settings that are especially important in ageing research.

The limited sample size and the gender imbalance—characterised by a higher proportion of female participants—restrict the generalisation of the findings. No formal power analysis was conducted, as the study was primarily descriptive. While the study highlights trends that contribute to the understanding of age-related gait adaptations, results should be interpreted with caution. Additionally, logistical constraints following data collection precluded further participant recruitment.

In addition, a younger adult control group was not included for direct comparison, as the primary aim of this work was to establish a population-specific descriptive profile for healthy older Portuguese adults in naturalistic settings. Recruiting a younger cohort was beyond the scope and resources of the project. To contextualise our findings, we compared them with published values from younger adult populations in studies using similar IMU-based systems and protocols, which we acknowledge may still introduce variability due to methodological differences. Future studies should incorporate directly measured younger controls, assessed with identical instrumentation, to strengthen interpretations of age-related gait adaptations.

Furthermore, recruitment was conducted exclusively through daycare centres, which may bias the sample toward lower-functioning older adults. This approach was chosen to ensure access to a safe and cooperative cohort for detailed gait assessments. Future studies should aim to include participants from more diverse living environments and activity levels to improve generalisability.

Future research should aim to recruit larger, more demographically balanced cohorts and incorporate complementary measurement modalities, such as force platforms and electromyography, and perform formal reliability and validation studies for gait event detection and pattern recognition algorithms, to achieve a more comprehensive biomechanical characterisation of gait changes in ageing populations.

## 4. Conclusions

All parameters were analysed using descriptive statistics, which allowed for a general characterisation of the sample under study. The results obtained and the conclusions drawn reflect only the average trend of the sample.

Regarding population characterisation, it was determined that the sample is overweight, and there is a tendency for men to have higher grip strength than women. In terms of the gait cycle analysis, symmetry in the walking pattern between the two lower limbs and between the two genders was observed. An increase in the stance phase and a corresponding decrease in the swing phase were noted. A significant increase in the double support phase was found in this sample.

With respect to the spatiotemporal parameters, reductions in walking speed, stride length, stride duration, and cadence were observed when compared to reference values for adults. An increase in stride width was noted. These differences indicate an adaptation by older adults to achieve a more stable and safer gait pattern.

For the kinematic parameters, a decrease in the mean peak plantar flexion angle and an increase in the mean peak dorsiflexion angle were observed when compared to adult reference values. Additionally, a decrease in the mean peak knee flexion angle and in peak hip extension angle was noted.

In conclusion, the lack of significance in some results does not imply that there are no differences in the studied group. However, the small sample size limits the ability to precisely characterise the ageing biomechanical parameters of the population.

This study presented the temporal/spatial and kinematic parameters of the gait pattern in a sample of 36 older adult volunteers. While IMU-based gait analysis in older adults is well-established, our results provide population-specific reference data for healthy older Portuguese adults and highlight both global age-related trends and region-specific biomechanical adaptations. These findings complement existing literature and may inform clinical assessment, rehabilitation strategies, and future research aimed at characterising gait changes in ageing populations.

## Figures and Tables

**Table 1:** Mean duration in seconds of the stance phase, swing phase, and gait cycle for the right and left lower limbs. Values represent overall gait cycle results across the study sample.

Limb	Right Limb	Left Limb
Stance phase (s)	1.9±0.5	1.8±0.4
Swing phase (s)	0.40±0.04	0.4±0.1
Gait cycle (s)	2.3±0.5	2.1±0.5

**Table 2:** Angular movement (°) for the hip, knee and ankle joints, for the left (a) and right (b) limbs. It is presented in terms of maximum peak plantar flexion angle and its location on the gait cycle (indicated in percentage of the cycle), and maximum peak dorsiflexion angle and its location on the gait cycle (indicated in percentage).

(a) Joint	Flexion	%GC	Extension	%GC
Left Hip	$28.1^{\circ }\pm 6.2^{\circ }$	48%	$-2.4^{\circ }\pm 5.0^{\circ }$	4%
Left Knee	$46.2^{\circ }\pm 7.4^{\circ }$	27%	$5.2^{\circ }\pm 5.1^{\circ }$	Begin of GC
Left Ankle	$-3.7^{\circ }\pm 7.1^{\circ }$ Plantar flexion	24%	$17.7^{\circ }\pm 6.3^{\circ }$ Dorsiflexion	4%
(b) Joint	Flexion	%GC	Extension	%GC
Right Hip	$30.0^{\circ }\pm 6.6^{\circ }$	98%	$-1.6^{\circ }\pm 5.2^{\circ }$	59%
Right Knee	$47.0^{\circ }\pm 9.9^{\circ }$	76%	$4.5^{\circ }\pm 6.2^{\circ }$	47%
Right Ankle	$-3.9^{\circ }\pm 5.0^{\circ }$ Plantar flexion	75%	$16.0^{\circ }\pm 4.4^{\circ }$ Dorsiflexion	58%

## Data Availability

Data supporting the reported results can be obtained by writing to anna.letournel@ess.ips.pt.
